# P53 and bcl-2 immunoexpression in patients with oral lichen planus and oral squamous cell carcinoma

**DOI:** 10.4317/medoral.18013

**Published:** 2012-05-01

**Authors:** Elba R. Leyva-Huerta, Constantino Ledesma-Montes, Rebeca E. Rojo-Botello, Elisa Vega-Memije

**Affiliations:** 1 Clinical and Experimental Pathology Laboratory. División de Estudios de Posgrado e Investigación. Facultad de Odontología, UNAM. México, D.F. MÉXICO; 2 Clinical Oral Pathology Laboratory. División de Estudios de Posgrado e Investigación. Facultad de Odontología, UNAM. México, D.F. MÉXICO. Member of the Cuerpo Académico. Facultad de Odontología. Universidad de Ciencias y Artes de Chiapas. Tuxtla Gutiérrez, Chiapas. MÉXICO; 3 Department of Dermatology. Hospital Manuel Gea González. México, D.F. MÉXICO

## Abstract

Objective: The aim of this study was to determine by immunohistochemistry the presence and significance of p53 and bcl-2 proteins in oral lichen planus (OLP) and oral squamous cell carcinoma (OSCC). Study Design: We used 21 cases diagnosed as OLP 16 diagnosed as OSCC and four normal gingival biopsies taken from healthy patients were used as controls. Slides were processed for immunohistochemistry using anti-p53 and anti-bcl-2 monoclonal antibodies. Results: We found p53 immunoexpression in 71.4% OLP cases and 68.7% OSCC cases, with no immunoexpression in control cases. Bcl-2 was negative for all OLP and OSCC cases, and mild positivity was observed in normal tissue. We found significant correlation among p53 expression and OSCC malignancy. Conclusions: Our results suggest that TP53 system mainly promotes a hyperproliferative state by cell cycle arrest of the OLP epithelial cells for repairing damaged DNA nor apoptosis and that anti-apoptotic action of bcl-2 is not important in this disease.

** Key words:**Oral lichen planus, oral squamous cell carcinoma, p53, Bcl-2, carcinogenesis, malignant transformation.

## Introduction

Lichen planus is a relatively common, chronic dermatologic disease affecting the oral mucosa. Most patients affected by lichen planus are adults and it has been reported that malignant transformation of oral lichen planus (OLP) varies from 0% to 2.1% (1-4). More than 90% of the malignant neoplasms of the oral cavity are squamous cell carcinomas of the lining mucosae and males are affected more often than females. In males, the country with the oral squamous cell carcinoma (OSCC) highest rate in the western world is currently France with elevated rates in some parts of Latin America (5).

Growth of keratinocytes is regulated by a delicate balance among molecules controlling cell survival such as bcl-2 and cell death as p53. bcl-2 is an anti-apoptotic membrane-associated molecule residing in the nuclear and mitochondrial membranes. It is inversely related to p53 since its expression prevents apoptotic cell death and its anti-apoptotic function is modulating the mito-chondrial release of cytochrome c (6).

Alternatively, the TP53 gene is a tumor suppressor gene that induces cell cycle arrest or apoptosis. p53 can exert its apoptotic functions inducing the expression of proteins involved in mitochondrial and death receptor-induced apoptotic pathways. In this fashion, p53 induces the expression of several mitochondrial proteins of the bcl-2 family and induces mitochondrial release of cytochrome c, which associates with Apaf-1 and caspase 9 to form an apoptosome. Moreover, Bax protein heterodimerizes with bcl-2, abrogates its function and promotes apoptosis (6).

The importance of both proteins in cell death and apoptosis in various oral diseases as OSCC, leukoplakia and other epithelial dysplasias and OLP has been the matter of intensive studies (6-9).

Many reports suggest that OLP is a potential pre-cancerous lesion and that both bcl-2 and p53 proteins can be involved in the malignant transformation of OLP to OSCC (2,4,10-20). Expression of both proteins in these lesions is not yet well established since conflicting results have been published (10-12,21-25).

Therefore, the aim of this study was to determine by immunohistochemistry the presence of p53 and bcl-2 in OLP and OSCC and their possible involvement in the process of cancer development.

## Material and Methods

This study was made in the Department of Dermatology of the Hospital General Manuel Gea González and in the Clinical and Experimental Pathology Laboratory of the División de Estudios de Posgrado e Investigación. Facultad de Odontología, UNAM, both located in Mexico City. Specimens were biopsy tissues from 37 patients with diagnosis of OLP and OSCC. A document of informed consent was previously signed and a complete clinical history was made to all patients. The protocol of this study was previously approved by the Ethics Committees of both institutions; five specimens from normal gingiva of clinically healthy subjects were also collected. OLP cases were graded as the WHO guidelines (26) in: reticular (r-OLP), erosive (e-OLP) and cases with reticular and erosive areas were classified as OLP mixed type (m-OLP). For OLP diagnosis, the clinico-pathological rules suggested by Al-Hashimi et al. (27) were used.

All specimens were fixed in 10% buffered formalin for 24 hours and routinely processed to obtain 5µ thick sections. H&E staining was used to confirm the original diagnosis, and to classify OSCC in low, intermediate and high grade.

Immunohistochemistry. Six deparafinized sections each case (three for each monoclonal antibody) were soaked in 10 mM/L, pH 6, citrate buffer and placed in a microwave oven during 10 min for antigen retrieval. Endogenous peroxidase was blocked using 0.3% H2O2 solution in methanol for five minutes at room temperature. Then, sections were rinsed in PBS for five minutes and covered with horse normal serum for 20 minutes at room temperature. Sections were incubated for 40 minutes at room temperature with monoclonal antibodies against p53 (NCI-953DO-7) and bcl-2 proteins (mouse clone 124), both antibodies were purchased from Dako (Carpinteria, CA). After washing in PBS, 2-3 drops of secondary antibody (Elite, PK-6102) were added and the sections were incubated for 20 minutes at room temperature. After two PBS rinses, all sections were counterstained with Mayer’s haematoxylin and mounted. Additional sections running in parallel but with omission of the primary antibodies, served as negative controls.

Specimens were examined microscopically to score p53 and bcl-2 staining intensity. Less than 5% of stained cells was graded negative (-); 6-25% positive cells was recorded as mild (+); 26-50% positive cells was scored as moderate (++) and more than 50% as intense (+++). Counting was made in not less than 100 cells per slide and a mean of positive cells obtained from all three analyzed slides per case was obtained. This analysis was performed by two well trained, previously calibrated Oral Pathologists (k=0.05). Student’s T test and Pearson’s correlation coefficient analysis were performed, p<0.05 was considered statistically significant.

## Results

Clinical findings. Twenty-one out of 37 patients were diagnosed with OLP. Patients’ age varied from 22 to 74 years (56+12.3 years). 76.2% were females, and tobacco and alcohol users corresponded to 28.6% and 14.3% respectively. In 14 cases (66.7%) OLP lesions involved oral tissues only; oral cavity and skin were 5 cases (23.8%); and oral cavity and genitals were 2 cases (9.5%). OLP lesions were located in buccal mucosa (n= 11; 52.4%), lips (n= 8; 38.1%), dorsal tongue and hard palate (n= 1; 6.3% respectively). Ten cases were classified as e-OLP, seven as m-OLP and four as r-OLP. 16 patients had OSCC; age range was from 33 to 93 years (66.8+14.4 years). 75% were male, 62.5% were tobacco users and 43.8% drank alcohol. OSCC cases were located as follows: labial mucosa (eight cases; 50%), lateral borders of the tongue (four cases; 25%), buccal mucosa (two cases; 12.5%), gingiva (one case; 6.3%), and another case involved the gingival and buccal mucosa (6.3%). Microscopically, 10 cases were classified as low grade, 5 as intermediate and one as high grade.

**Figure 1:**
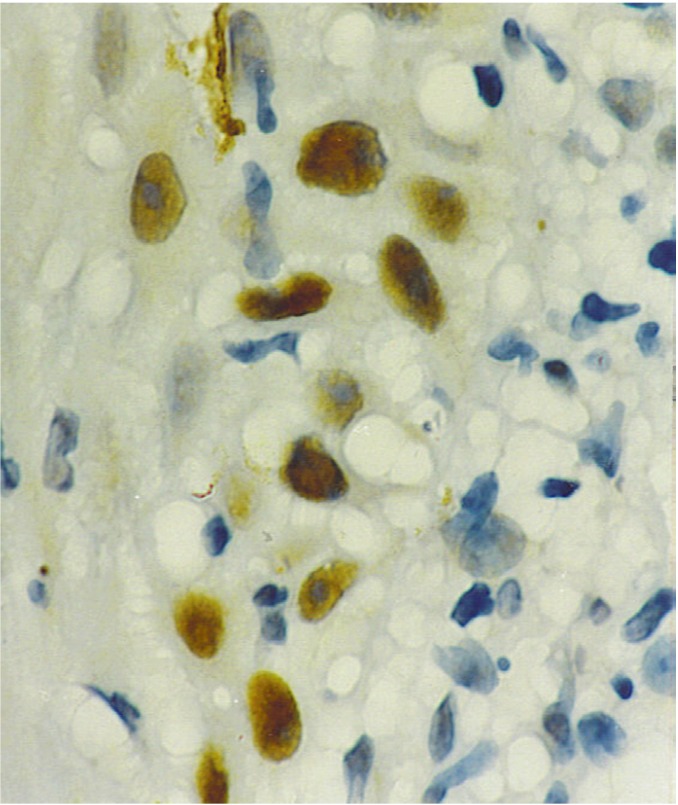
Photomicrograph showing intense p53 immunopositivity in lichen planus. p53 immunostaining technique. 400X.

p53 Immunoexpression. Immunoreactivity was observed as a brownish staining in the cellular nucleus (Fig. 1), it was negative in the normal tissues and six OLP cases, mild in 10, moderate in three and intense in two cases. Both cases with intense p53 expression were e-OLP type. ( Table 1) shows p53 immunoexpression according to OLP type. No statistical significance was found among p53 expression and gender, age, alcohol or smoking habits, and location of the lesions (p>0.05). Statistical significance was found only among p53 expression and clinical types of OLP (p< 0.02).

**Table 1:**
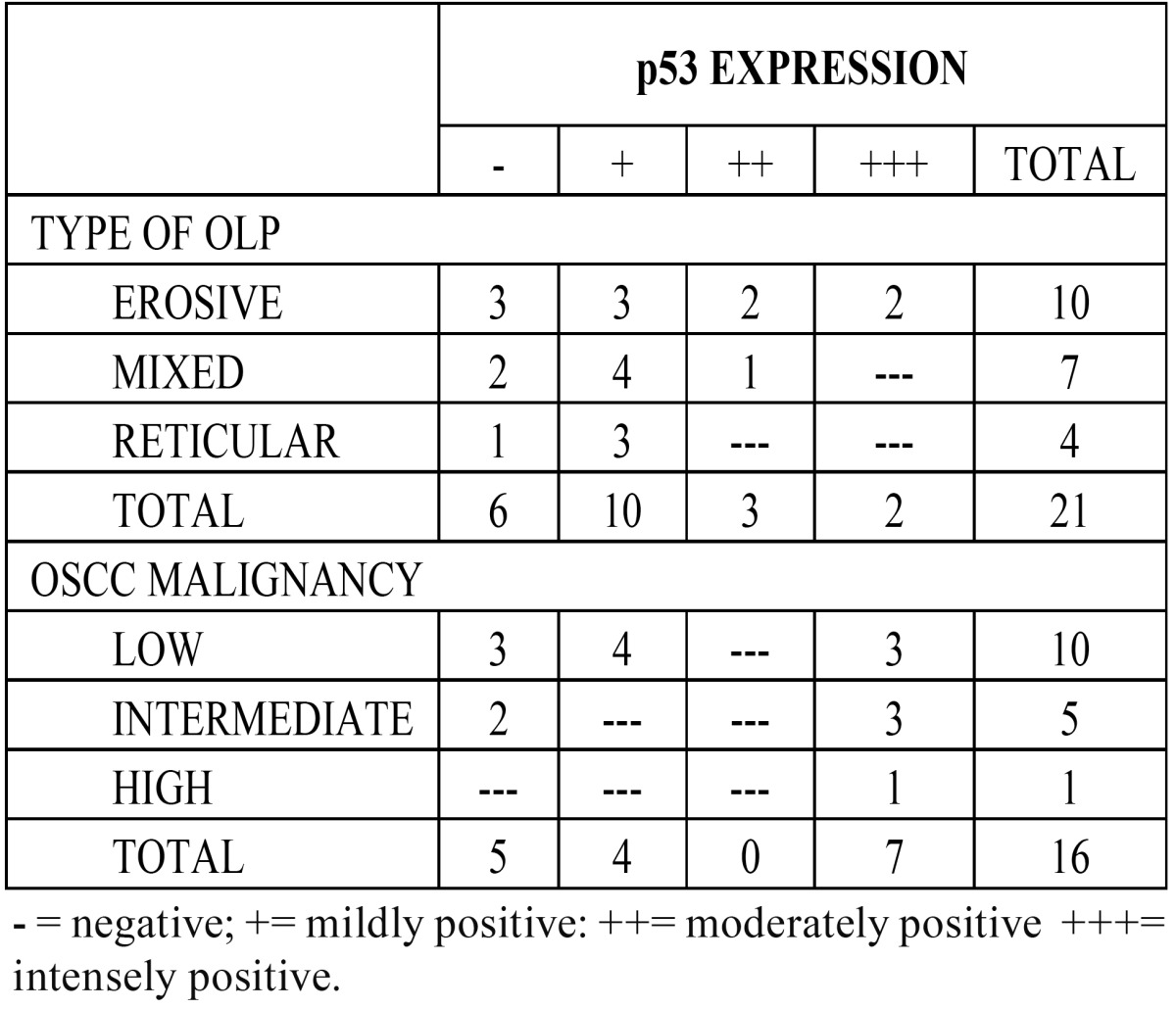
p53 Immunoexpression In Studied Cases.

**Figure 2:**
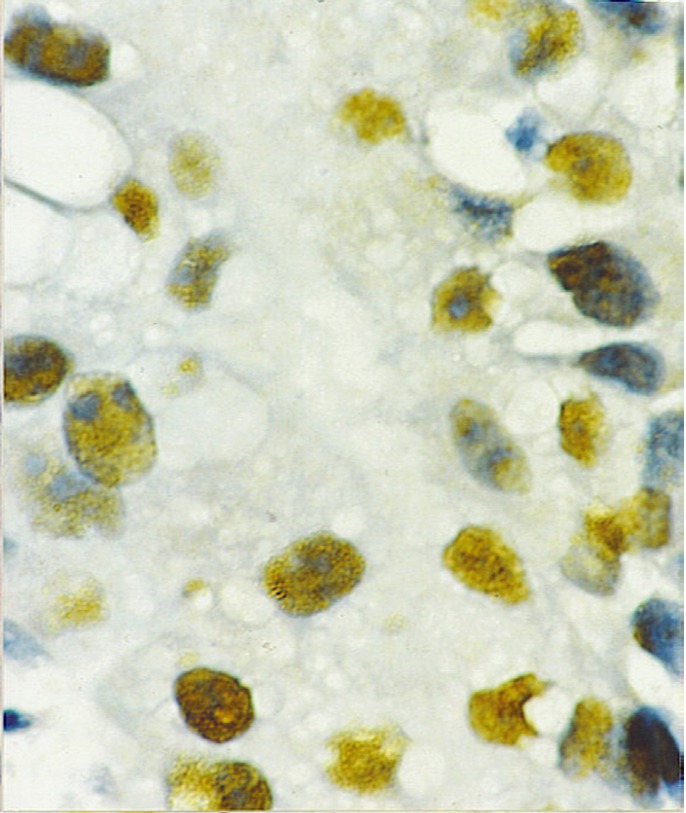
Intense p53 immunoexpression in an oral squamous cell carcinoma. p53 immunostaining technique. 400 X.

p53 expression was negative in 5 OSCC cases (31.2%); mild in 4 (25%) and intense in 7 cases (43.8%). ( Table 1) shows p53 immunoexpression according to OSCC malignancy. Mild expression predominated in cases of low grade malignancy; and the only case classified as high grade showed intense expression of p53 (Fig. 2). Normal tissue did not show p53 expression. We found statistically significant correlation among p53 expression and the degree of malignancy of the studied cases (p<0.03). When we analyzed the correlation among gender, age, smoking habit, alcoholism and location of the OSCC cases with p53 immunoexpression, no statistically significant differences were found (p>0.05). There was statistical difference among p53 immunoexpression in OSCC and OLP cases. (p<0.001).

**Figure 3:**
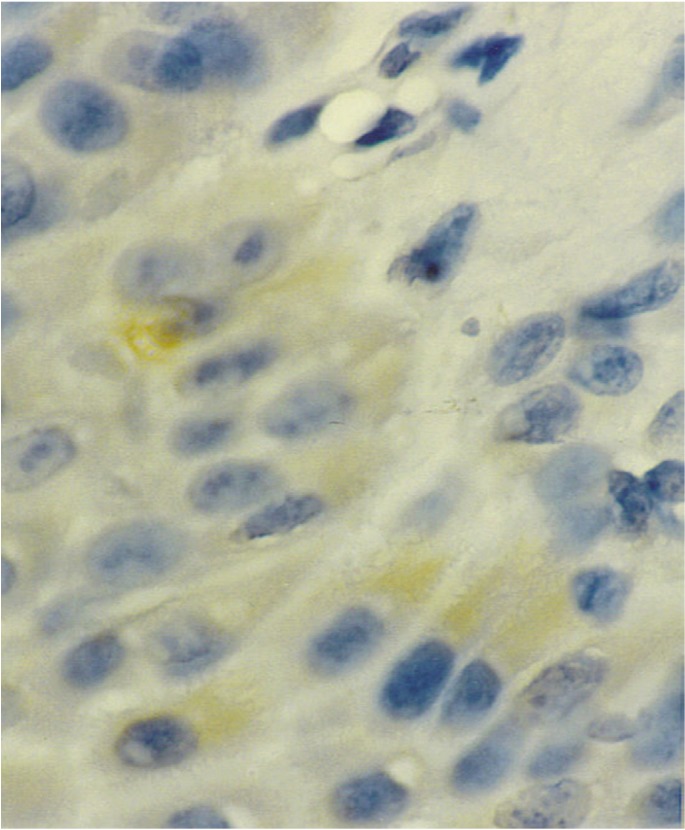
Normal tissue showing mild immunoexpression to bcl-2. 400 X.

Bcl-2 immunoexpression. It was negative in all OLP and OSSC cases. In normal tissue a mild positivity was seen in the cytoplasm of the basal cells (Fig. 3).

## Discussion

Despite there are not factors clearly associated with malignant transformation to oral lichen planus. OLP has been regarded as a premalignant condition; this premalignant potential has been demonstrated by the results in previously published studies (2-4). This implies that a patient with OLP has an increased risk for developing OSCC, but not necessarily in an existing OLP lesion.

p53 and bcl-2 have been related to malignant transformation of oral epithelial cells (6-9,23,25). It has been considered that the long term permanence of OLP in the oral cavity is probably the factor that triggers its malignant transformation and it is specially risky with e-OLP and if it stays for more than five years (2-4,27).

p53 immunoexpression was reported in OLP (10-12,14,15,19,21,23) and in our study, 71.5% of OLP cases were positive. Out of these cases, 7 were of e-OLP and two of them showed intense positivity. p53 expression in OSCC has been reported in up to 81% of the cases (7,21,23). In this study, 68.8% of our OSCC cases were p53 positive; and this figure is consistent with the above mentioned studies. Our results and those previously reported (7-9) indicate that p53 expression is related with the malignant degree of OSCC. In contrast, other reports found no correlation (12,13,28).

We failed to demonstrate the value of p53 as a predictor for OLP transformation to OSCC since 15/21 OLP studied cases showed p53 positivity. These results suggest that may be p53 is an inaccurate tool for predicting OLP malignant transformation. This suggestion is supported because the majority of the OLP studied cases were positive to p53 and this prediction value is challenged because it is reasonable to assume that the majority of these p53-positive cases will never transform to OSCC. In our study, we found that p53 expression rate in OSCC and OLP were similar (68.8% and 71.5% respectively), these results may suggest there are another cellular mechanisms involved in transformation of OLP to OSCC.

There has been proposed a hypothesis that malignant transformation is a consequence of alterations in cell cycle control affecting extensive areas of the oral mucosa (14). Previous results (10,14,15,29,30), indicate that apoptosis is of scant importance in the disease, and that many damaged basal epithelial cells respond to aggression by increasing their proliferation rate (15-17). Recent findings suggest that this hyperproliferative state constitutes a form of cell response attempting to preserve the epithelial structure avoiding the appearance of ulcerations (15,18). However, the malignant transformation rate of OLP is low and as it was previously suggested, this low rate of malignant transformation in OLP is due to activation of the TP53 system, which exerts priority action to promote the repair of damaged DNA (15,18). In this context, the cases of malignization could correspond to situations where mutations or other inactivating mechanisms prevent the TP53 system from acting correctly and this in turn possibly constitutes a key element for initiation of the malignant transformation process.

A number of authors estimate that TP53 overexpression constitutes a form of cell response to the hyperproliferative state frequently seen in OLP (17,19,20). González-Moles et al. results (18) suggested a significant association among p53 and Ki-67 expression, and they did not accept that the epithelium associates cell proliferation (Ki-67 positive cells) and cell cycle arrest/apoptosis (p53-positive cells).

González-Moles et al. (18) concluded that in OLP, the TP53 system is frequently activated fundamentally to arrest the cell cycle and to improve DNA repair, since they observed no association among p53 and caspase-3 expression. As it was previously reported (15,18,30), this form of response to aggression with low apoptosis, frequent wild p53 expression and increased cell proliferation rate constitutes a molecular mechanism designed to preserve the epithelium in OLP to avoid ulcerations. Recent observations reinforce this hypothesis because the lack of association found among p53 and Ki-67 expression (18). These results suggest that the hyperproliferative state of OLP is not secondary to TP53 mutation.

It is possible that p53 mutations may constitute an important oncogenic event in malignantly transformed OLP, with generation of a new cell population with summative oncogenic events which led to acquisition of a malignant phenotype. Thus, the low frequency of p53 mutations could explain the low malignization rate of OLP (18). It is possible that p53 over expression in the erosive type of OLP can not be related to its possible malignant transformation to OSCC, since only 23.8% of the OLP cases analyzed in this study showed moderate to intense immunoexpression to this protein.

Previously reported findings regarding bcl-2 expression in OSSC are controversial (10-13,23-25,27). Jordan et al (27) reported cytoplasmic expression in 60% of their studied OSCCs suggesting that bcl-2 expression in poorly differentiated OSCC cases was due to malignant keratinocyte inability to reach final differentiation, maintaining a bcl-2 phenotype of the mother cell. Two re-ports (22,28) demonstrated an inverse correlation among p53 and bcl-2 expression in OSCC suggesting that one of these proteins could substitute the other during carcinogenesis and that TP53 gene can downregulate the bcl-2 gene during the apoptotic process. Our results support this point of view since all our OSCC cases were negative for bcl-2 protein.

As it was in other studies (11,22,28), our results show that there were no bcl-2 expression in the OLP studied cases. Results from these studies suggest that bcl-2 protein does not seem to be involved in the OLP epithelial changes, since no immunoreactivity was seen; these results also suggest that loss of the bcl-2 anti-apoptotic control is associated with a concomitant loss of other pro-survival molecules or an increase in the pro-apoptotic molecules in OLP. It is also possible that a combination of long term survival of the bcl-2 negative OLP cells and strong p53 immunoexpression could account to increase the potential to malignant transformation in e-OLP cases. In contrast Tanda et al. suggested that the absence of low rate of apoptosis in OLP is a consequence of the anti-apoptotic action exerted by bcl-2 (11).

Data concerning bcl-2 expression in OSCC is not uniform, with positivity varying from 23 to 100% (8,9,22). It is interesting to note, that contrasting to several studies, all our OSCC cases were negative for bcl-2. This disparity in bcl-2 expression from different studies may reflect subtle inherent differences in upstream genetic events among different populations, gender and age of the patients, anatomical location of the studied lesions and possibly differences in the environmental, hygienic and dietary conditions.

It is possible that alterations in mutation of the TP53 and bcl-2 systems may play an important role in the development of OSCC probably by allowing the neoplastic cells to escape from apoptosis. In summary, both lichen planus and OSCC show similar expressions for p-53 and bcl-2, and it is possible that alterations of these genes may be relevant in the long term transformation process of OLP to OSCC. There are other mechanisms potentially involved in the OLP change to OSCC. If the apoptotic phenomenon is of little importance in OLP (14,15), this is a surprising conclusion in an epithelium subjected to an intense and persistent autoimmune aggression. They propose that OLP cells respond more frequently with arrest or senesce than with apoptosis. It is also possible that the action by different oncogenic insults on proliferating OLP cells may play a role in the transformation of OLP to OSCC (1-4,14).

The results of our study and those from others (14,15) agree in that it may be a consequence of alterations in the cycle cell control mechanisms affecting wide areas of the oral mucosa and that the low rate of apoptosis and the increased rate of the OLP affected epithelium will serve to maintain the epithelial architecture that may underline a predisposition to developing cancer in OLP affected tissues and that malignant transformation would presumably be favored by a failure of the TP53 system permitting that proliferating cells do not enter to apoptosis and that they are under the TP53 system and that these events could led to accumulation of a malignant cell phenotype.
